# Delayed Contrast Enhancement Imaging of a Murine Model for Ischemia Reperfusion with Carbon Nanotube Micro-CT

**DOI:** 10.1371/journal.pone.0115607

**Published:** 2015-01-30

**Authors:** Laurel M. Burk, Ko-Han Wang, John Matthew Wait, Eunice Kang, Monte Willis, Jianping Lu, Otto Zhou, Yueh Z. Lee

**Affiliations:** 1 Department of Physics and Astronomy, University of North Carolina at Chapel Hill, Chapel Hill, NC 27599, United States of America; 2 Department of Radiology, University of North Carolina at Chapel Hill, Chapel Hill, NC 27599, United States of America; 3 Division of Cardiology, McAllister Heart Institute, University of North Carolina at Chapel Hill, Chapel Hill, NC 27599, United States of America; 4 Curriculum in Applied Science and Engineering, University of North Carolina at Chapel Hill, Chapel Hill, NC 27599, United States of America; University Francisco de Vitoria School of Medicine, SPAIN

## Abstract

We aim to demonstrate the application of free-breathing prospectively gated carbon nanotube (CNT) micro-CT by evaluating a myocardial infarction model with a delayed contrast enhancement technique. Evaluation of murine cardiac models using micro-CT imaging has historically been limited by extreme imaging requirements. Newly-developed CNT-based x-ray sources offer precise temporal resolution, allowing elimination of physiological motion through prospective gating. Using free-breathing, cardiac-gated CNT micro-CT, a myocardial infarction model can be studied non-invasively and with high resolution. Myocardial infarction was induced in eight male C57BL/6 mice aged 8–12 weeks. The ischemia reperfusion model was achieved by surgically occluding the LAD artery for 30 minutes followed by 24 hours of reperfusion. Tail vein catheters were placed for contrast administration. Iohexol 300mgI/mL was administered followed by images obtained in diastole. Iodinated lipid blood pool contrast agent was then administered, followed with images at systole and diastole. Respiratory and cardiac signals were monitored externally and used to gate the scans of free-breathing subjects. Seven control animals were scanned using the same imaging protocol. After imaging, the heart was harvested, cut into 1mm slices and stained with TTC. Post-processing analysis was performed using ITK-Snap and MATLAB. All animals demonstrated obvious delayed contrast enhancement in the left ventricular wall following the Iohexol injection. The blood pool contrast agent revealed significant changes in cardiac function quantified by 3-D volume ejection fractions. All subjects demonstrated areas of myocardial infarct in the LAD distribution on both TTC staining and micro-CT imaging. The CNT micro-CT system aids straightforward, free-breathing, prospectively-gated 3-D murine cardiac imaging. Delayed contrast enhancement allows identification of infarcted myocardium after a myocardial ischemic event. We demonstrate the ability to consistently identify areas of myocardial infarct in mice and provide functional cardiac information using a delayed contrast enhancement technique.

## Introduction

The leading cause of mortality worldwide is cardiovascular disease. The World Health Organization’s most recent reports estimate that a total 17.3 million people died in 2008 as a result cardiovascular disease, or 30% of all global deaths, with 7.3 million attributed specifically to coronary heart disease [1]. Developments in diagnosis and treatment of cardiac illness rely heavily on murine models of human disease, yet due to the simultaneous demands of high spatial and temporal resolutions imposed by small size and rapid heart rates, there are limited imaging tools with which researchers are able to evaluate small animal cardiac structure and function. Due to speed, simplicity, ubiquity, and low cost, the current most widely employed modality for *in-vivo* murine cardiac imaging is echocardiography. Yet there are significant limitations to this approach: 2-D ultrasound images cannot easily distinguish between nonfunctional infarct, ischemic, stunned, hibernating, and healthy myocardium, as these differences are largely functional rather than structural on the length scales visible with available *in-vivo* murine imaging modalities. The current gold standard for determining myocardial tissue viability in the clinical setting is magnetic resonance imaging (MRI) with delayed contrast enhancement, which combines the ease and high spatial resolution of structural imaging with a contrast administration protocol that illuminates necrotic myocardial tissue [2]. Infarction of the myocardium caused by obstruction of the coronary arteries results in non-viable tissue. In the presence of subsequent reperfusion, edema may occur with an associated increase in permeability of the capillaries. This loss of membrane integrity results in contrast agent retention within necrotic regions of the myocardium and a characteristic delayed enhancement effect in these regions after intravenous administration of the agent.

Delayed contrast imaging is made possible because the gadolinium-based vascular contrast agents used in MR imaging distribute themselves in the extracellular spaces of healthy myocardium soon after intravenous administration, while these agents are too large to be admitted into the normal myocardial cells. However, the cellular changes resulting from ischemia affect arterial flow rate and the permeability of the capillaries. Within necrotic and acute infarcted regions of myocardium, a loss of membrane integrity allows contrast agent molecules to leak from capillaries into these regions, and contrast is retained in these tissues past the typical wash-out time in uninjured extracellular spaces. The result is a characteristic delayed enhancement in necrotic regions of myocardium captured in MR images acquired during an appropriate time window during which gadolinium has leeched into infarcted tissue but has not yet leeched out again. This phenomenon has already been successfully demonstrated in murine MRI imaging studies [3]. The same mechanism of contrast medium leakage in infarcted tissue applies to many iodinated blood contrast agents used in CT whose particles are of approximately the same size as the gadolinium agents of MRI [4, 6].

Surgically-induced ischemia and reperfusion of the LAD is also expected to significantly inhibit left ventricular myocardial function, decreasing the heart’s ability to contract and pump blood. Any change in cardiac output can be quantified by a comparison between the ejection fractions of subjects, which have undergone ischemia and reperfusion and the ejection fractions of control subjects. When blur-free micro computed tomography (micro-CT) images can be obtained from live murine subjects during diastolic and systolic cardiac phases, accurate ejection fractions can be calculated as a metric of left ventricular function. Thus, high quality cardiac micro-CT, when achieved, provides both structural and functional information about the murine heart following ischemia and reperfusion.

In general, the ability to acquire high quality murine cardiac images using traditional micro-CT demands a high temporal resolution, which is challenging to achieve, since subjects experience rapid cardiac and respiration rates. Full scan times of 10–15ms would be necessary to complete a full, motion-free CT scan during a single systolic phase, but this is not possible with current technology due to gantry speed and x-ray tube flux limitations. Instead, physiological gating is necessary for in vivo cardiac imaging of mice, acquiring x-ray projection images only during the phase of interest over several successive cardiac cycles. Retrospective gating techniques allow for rapid scan times but involve several gantry rotations per scan and subsequently deliver a higher radiation dose to the subject [5]. A typical prospectively-gated cardiac micro-CT does not deliver an extra patient dose, but it can require a complex protocol including intubation and ventilation of the subjects to synchronize x-ray projections to the desired respiratory and cardiac phase, as well as continuous IV contrast agent infusions [6].

Delayed iodine contrast enhancement of myocardial tissue in murine models has previously been demonstrated by Nahrendorf, *et al*. and in small animal studies using micro-CT [4]. Though this previous work was successful in demonstrating the validity of the delayed contrast enhancement protocol in murine micro-CT imaging, the selected protocol required that subjects be intubated for mechanical ventilation, a technique which carries certain risks to pulmonary health [5] and which challenges longitudinal studies of individual subjects. All of the previous studies on small animal in vivo imaging of myocardial infarction have employed CT scanners with traditional thermionic x-ray sources, and involve all of the previously stated limitations.

Many of the difficulties of traditional *in-vivo* micro-CT are overcome with the development of a micro-CT with carbon nanotube (CNT) cathode [8]. The device’s cold cathode operates through field emission rather than the thermionic emission of traditional x-ray tubes, resulting in several benefits. Because field emission is controlled through modulation of an electric field at the surface of the carbon nanotube cathode, switching is triggered in a fraction of a millisecond and steady exposures as short as 10ms can be achieved, both of which are sufficient for blur-free murine cardiac imaging [9]. By reducing cathode heat concerns, sources can be made compactly, and many x-ray emitters may be combined into arrays of various sizes and shapes [10, 11]. X-ray pulse responsiveness is rapid and periodicity is not required, so non-invasive cardiac imaging of free breathing animals can easily be performed with such a source using prospective physiological gating [12]. In the micro-CT imaging device utilized for this study, a single-cathode x-ray source was operated in a prospectively-gated protocol simultaneously synchronizing pulses to the semi-periodic predetermined phases of the cardiac and respiratory cycles. Thus the CNT micro-CT system eliminates the need for intubation and its associated risks and is robust with respect to small fluctuations in the respiratory and cardiac rates over the course of a scan.

Here, we evaluate a murine model for acute myocardial infarction using the CNT micro-CT and a delayed contrast enhancement technique with an iodinated contrast agent administered in a bolus. The surgical model for ischemia and reperfusion was produced by surgical occlusion of the left anterior descending artery (LAD) with a suture. Ischemia was maintained for thirty minutes and was followed by twenty four hours of reperfusion prior to obtaining CT images. Two different iodine-based contrast media were administered to the subjects via tail vein catheter. Iohexol 300 mg/mL (Omnipaque 300, GE Healthcare) was chosen to demonstrate the delayed contrast effect due to its rapid distribution and quick wash-out. Fenestra VC (Art Advanced Research Technologies, Inc, Montreal, Canada) [13], with different molecular properties and a longer half-life, was then administered to highlight the blood pool and left ventricle (LV) volumes in order to evaluate cardiac output. In CT images acquired over multiple time-points following IV contrast administration and during both the systolic and diastolic phases, the anticipated structural and functional changes resulting from LAD obstruction were observed.

## Materials and Methods

### 1. Disease Model

This study was carried out in strict accordance with the recommendations in the Guide for the Care and Use of Laboratory Animals of the National Institutes of Health. The protocol was approved by the Institutional Animal Care and Use Committee of the University of North Carolina at Chapel Hill (IACUC ID: 09-340.0). All surgeries were performed under sodium pentobarbital anesthesia, and all micro-CT imaging and contrast administration was performed under vaporized isoflurane anesthesia.

Myocardial infarction was induced in eight C57BL/6 male mice aged 8–12 weeks (∼30g) (Jackson Laboratories) one day prior to the imaging study. The ischemia reperfusion model was achieved by surgically occluding the LAD artery for 30 minutes followed by 24 hours of reperfusion [14]. In summary: subjects were anesthetized with 45 mg/kg pentobarbital, intubated, and ventilated with 100% oxygen. The chest cavity was opened by an incision of the left fourth intercostal space, and a 7–0 silk suture was passed underneath the LAD artery 1 to 2 mm below the left auricle and tied around a 1-mm length of polyethylene tubing. After 30 minutes of occlusion, blood flow was restored and the chest wall was closed.

Tail vein catheters were placed for contrast administration immediately prior to imaging. During the imaging procedure, subjects were anesthetized using isoflurane vapor in medical grade oxygen (initial dose at 2.5% followed by a continual dose of 1–1.5%, adjusted as necessary to maintain constant respiratory and cardiac rates for all procedures). Animals breathed freely throughout the entire imaging procedure, preventing possible complications that could arise from intubation and forced ventilation [7]. To facilitate the minimally-invasive imaging protocol, a custom imaging cradle was designed to interface with a small bellows-type pneumatic respiration sensor for tracking abdominal and organ motion. The cradle was manufactured with acrylonitrile butadiene styrene plastic in a 3-D printer, and subjects were positioned prone during imaging so that the abdomen rested comfortably atop the bellows sensor. Cardiac signals were monitored via electrocardiography (ECG) leads affixed to the paws (Soft Cloth Pre-wired Neonatal Radiolucent Electrode, 3M, St. Paul, MN). Analog signals generated by the respiratory pressure sensor / transducer and by ECG were input into a commercial patient monitoring system (Biovet, m2m Imaging Corp, Cleveland OH) and were used to trigger prospective physiologically-gated imaging.

### 2. Imaging Protocol

The CT imaging of this study was performed on a custom-built cone beam micro-CT system which consists of a field-emission x-ray source and a flat panel detector, both mounted on a step-and-shoot rotating gantry [12]. The x-ray source contains a cathode constructed from CNTs which emit electrons via field emission when an electric field is applied, two static Einzel-type focusing structures, and a stationary tungsten anode target. The system’s camera is a cesium iodine flat-panel detector (C7940-DK, Hamamatsu Corp, Hamamatsu Japan) with 50 micron pixels and a 12 × 12 cm field of view.

**Figure 1:**
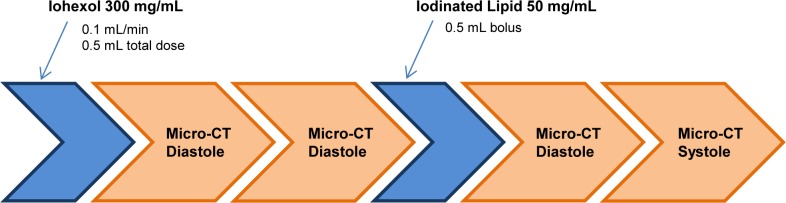
The contrast administration and imaging protocol. Four micro-CT images were acquired using two iodinated contrast agents, Iohexol 300 mg I/mL and Fenestra VC. Images were acquired during either diastole (on r-wave) or systole (55 msec delay from r-wave). Acquisition of each gated micro-CT image required 10 to 15 minutes. After successful completion each stage of the protocol, the next immediately commenced.

All CT images were acquired during the end exhalation phase of respiration and with a 0 or 55 msec delay following the R-wave, corresponding to the diastolic or systolic cardiac phases, respectively. The protocol, including contrast administration and image acquisition times, is displayed in Fig. 1. Iohexol at a concentration of 300 mg of iodine per 1 mL of solution (300 mg I /mL) was administered at a dose of 0.1 mL/5 g body weight, followed by two CT acquisitions triggered on the R-wave. These CT images were obtained to show delayed contrast enhancement in the infarcted regions of the myocardium, and they occurred at approximately 5 and 15 minutes after contrast administration to determine optimal time delay. Fenestra VC was then administered at a 0.1 mL/5 g dose, followed by images acquired at 0 and 55 msec after the R-wave. This second pair of images was used to compare ventricular volumes in diastole and systole and to calculate changes in ejection fraction caused by ischemia and reperfusion.

7 wild-type mice were also imaged in a similar protocol as controls. Physiologically-gated micro-CT images of these animals were obtained during the diastolic and systolic cardiac phases after administration of the iodinated lipid blood pool agent. These images were used as a baseline comparison for ejection fraction measurements.

Images were acquired in a step-and-shoot protocol with 286 projections over a total of 200 degrees gantry rotation. Each x-ray projection exposure was 15 msec in duration with 50 kVp energy and a 3 mA cathode current. The system control software was written in Labview (National Instruments Corp, Austin, Texas, US) and used to synchronize the flat-panel detector, operating on a 1 Hz fixed frame rate, and the x-ray source with the physiological trigger signals. The absolute minimum scan time of the system is 286 seconds to allow for gantry rotation and image readout. Synchronization requirements imposed by a physiologically-gated protocol increase the scan time to an approximate minimum of ten minutes per scan, although the exact scan duration is dependent upon individual cardiac and respiration rates.

### 3. Image Processing and Analysis

After acquisition, projections were preprocessed with a script in MATLAB (MATLAB and Imaging Processing Toolbox, The MathWorks, Inc., Natick, Massachusetts, US) to eliminate defective detector pixels and lines and then reconstructed to a 3-D volume with commercial reconstruction software (COBRA, Exxim Computing Corporation, Pleasanton, CA). As a part of 3D reconstruction, Hounsfield unit correction was performed to normalize to known attenuations of water and air. Scanner resolution after reconstruction was 77 μm isotropic.

Using a partially-automatic segmentation algorithm based on volume-growing region competition snakes in the Dicom viewer ITK-SNAP [15], micro-CT images enhanced with the iodinated lipid blood pool contrast agent were analyzed to measure LV blood pool volumes. A user-defined threshold HU range allowed the program to distinguish between myocardial tissue and the blood pool of the LV. Small seed volumes were manually within the LV, and ITK-Snap propagated these volumes via snake evolution until the entire ventricle was filled. After segmentation, LV volumes were recorded in units of mm^3^.

CT estimates of LV volumes are commonly used in the clinic for calculation of ejection fraction [16]. Using high-resolution and low-blur murine cardiac micro-CT images, the same method can be used to obtain ejection fractions from mice [4]. The calculation of ejection fraction for each subject was performed with the measured LV volumes for diastole and systole, V_Dia_ and V_Sys_, as EF = (V_Dia_ − V_Sys_)/V_Dia_.

A MATLAB program was written to measure infarcted tissue volumes from the reconstructed 3-D image sets of the first Iohexol-enhanced CTs acquired for each subject. HU thresholds were defined in order to segment and measure the total volumes of infarct and of the LV myocardium. Infarct sizes are reported as a percentage of the total LV myocardium for best comparison with histology.

### 4. Histology

After imaging, subjects were sacrificed and their hearts were cut into 1mm slices and stained with triphenyl tetrazolium chloride (TTC) to visualize uninjured and necrotic tissues. Slices were digitally photographed (both front and back) with an Olympus DP71 digital camera (Olympus Corporation, Center Valley, Pennsylvania USA) with 200 ISO speed and 0.71 s exposure time. The resulting photographs were stored in jpeg format with dimensions of 1360 × 1024 pixels and 144 dpi.

A MATLAB program was written to analyze each digitized histological slice to segment and measure the areas of myocardium with negative TTC stain uptake corresponding to infarcted tissue. The calculated infarct volume was reported as a percentage of total myocardium volume for each subject to eliminate the effects of tissue shrinkage, and a statistical comparison was made with the percent infarct volume obtained from CT images.

## Results

All eight animals survived the surgery, and all survived the delayed enhancement imaging portion of the protocol. One animal with the most extensive damage to the myocardium did not survive to be imaged for the lipid blood pool agent imaging and ejection fraction measurements. High quality CT images were obtained for all surviving animals. During CT acquisition, the mean respiration rate of the ischemia reperfusion model mice was 108 ± 17 bpm and the mean cardiac rate was 420 ± 70 bpm. Obvious delayed contrast enhancement following Iohexol administration was seen in the LV wall in CT images for all subjects. The blood pool contrast agent revealed changes in cardiac function which were quantified by low ejection fractions. All subjects demonstrated areas of myocardial infarct in the LAD distribution in reconstructed CNT micro-CT images; these regions corresponded with the areas lacking TTC stain uptake in the histological results.

**Figure 2:**
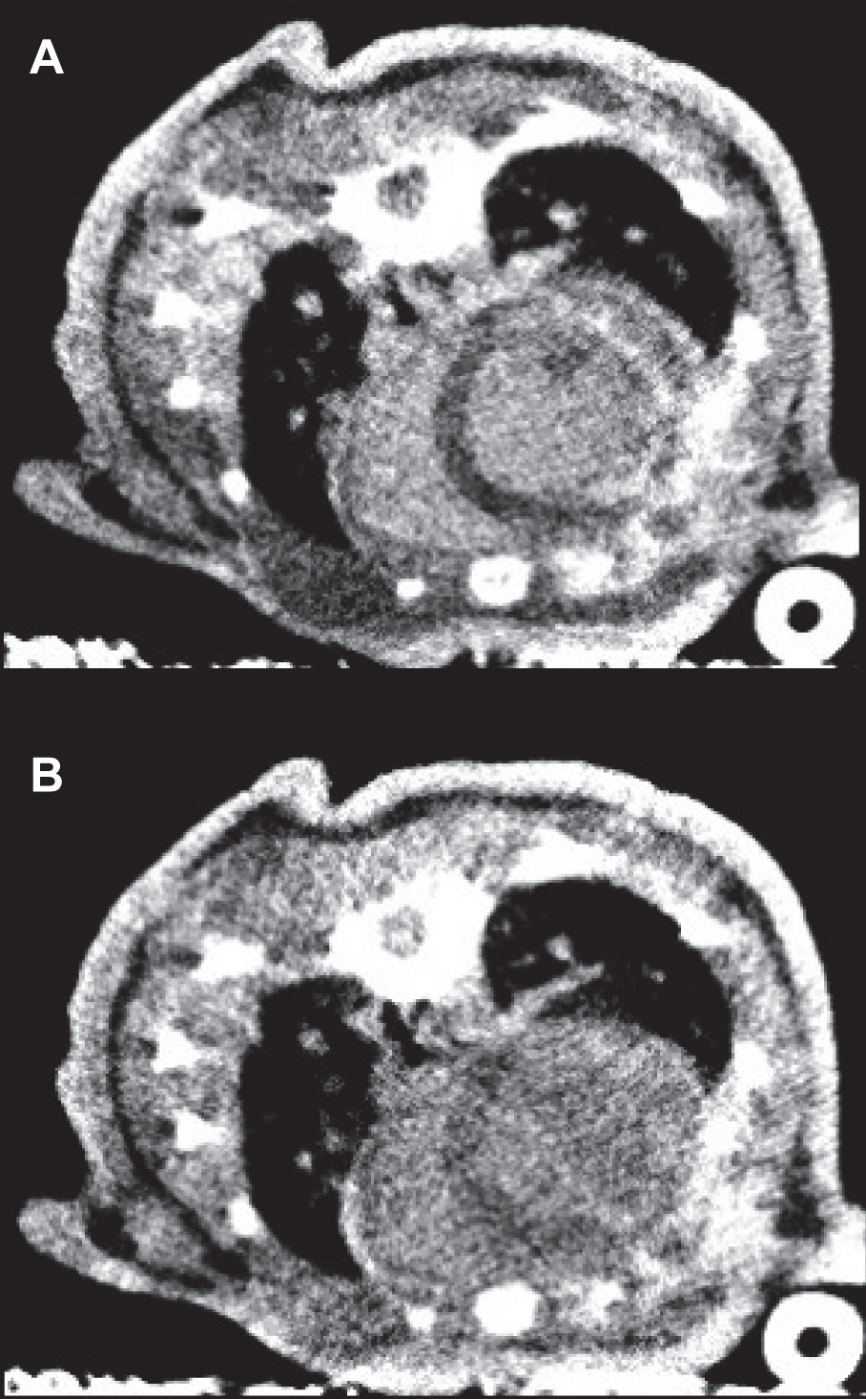
Micro-CT images of the ischemia reperfusion murine model. Images acquired an average of 13 (a) and 30 (b) minutes after administration of Iohexol display delayed contrast enhancement within regions of infarcted tissue.

Axial cross-sections of the micro-CT images for a representative subject are displayed in grayscale in Fig. 2. Images acquired an average of (A) thirteen and (B) twenty-five minutes after administration of the short half-life iodine contrast agent show hyper-enhancement within the myocardial wall which corresponds to the region most at risk of ischemia when the left anterior descending artery is obstructed. Delayed enhancement is visible at both time points, indicating that the optimal time window for visualizing infarcted tissue lies somewhere in the range of 5–30 minutes after administration and is not limited by the cardiac-gated micro-CT scan time, 10–20 min for a single phase.

**Figure 3:**
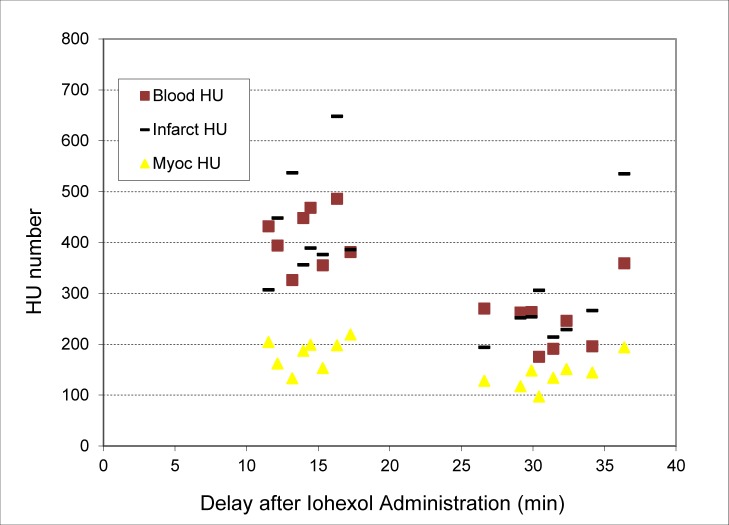
CT contrast (in Hounsfield units) with Iohexol for materials-of-interest plotted over time. During the first image acquisition (average 13 minutes after administration), CT numbers for the blood, infarcted region, and myocardium were 411 ± 56 HU, 431 ± 111 HU, and 182 ± 29.4, respectively. During the second image acquisition (average 30 minutes after administration), CT numbers for the blood, infarcted region, and myocardium were 245 ± 59, 281 ± 108, and 139 ± 28, respectively.

CT numbers within regions of interest comprised of the blood pool, myocardium, and infarcted tissues were measured from the first two CT images acquired of each subject. They are plotted as HU over time in Fig. 3. During the first image acquisition (average 13 minutes after administration), CT numbers for the blood, infarcted region, and myocardium were 411 ± 56 HU, 431 ± 111 HU, and 182 ± 29, respectively. During the second image acquisition (average 30 minutes after administration), CT numbers for the blood, infarcted region, and myocardium were 245 ± 59, 281 ± 108, and 139 ± 28, respectively.

Delayed hyper-enhancement occurs in all imaging time-points following the administration of Iohexol but is strongest during the first image acquisition. While the CT numbers for blood and infarct are similar in many of the images (411 ± 56 HU and 431 ± 111 HU during the first image acquisition), the two are easily distinguishable within the context, since the infarct region is always imbedded within the myocardial wall. Both blood and infarct are clearly distinguishable from myocardium in all Iohexol-enhanced images (p < 0.0001) during the first of the two imaging time points. Because of the acquisition time for physiologically-gated micro-CT protocols (∼10–20 minutes), the initial wash-in portion of delayed enhancement could not be seen. However, the first CT image, acquired an average of 13 minutes after contrast injection, was optimal for visualizing hyper-enhancement of the infarcted region. If acquired within the appropriate time window, only one image is required to visualize the infarcted regions.

**Figure 4:**
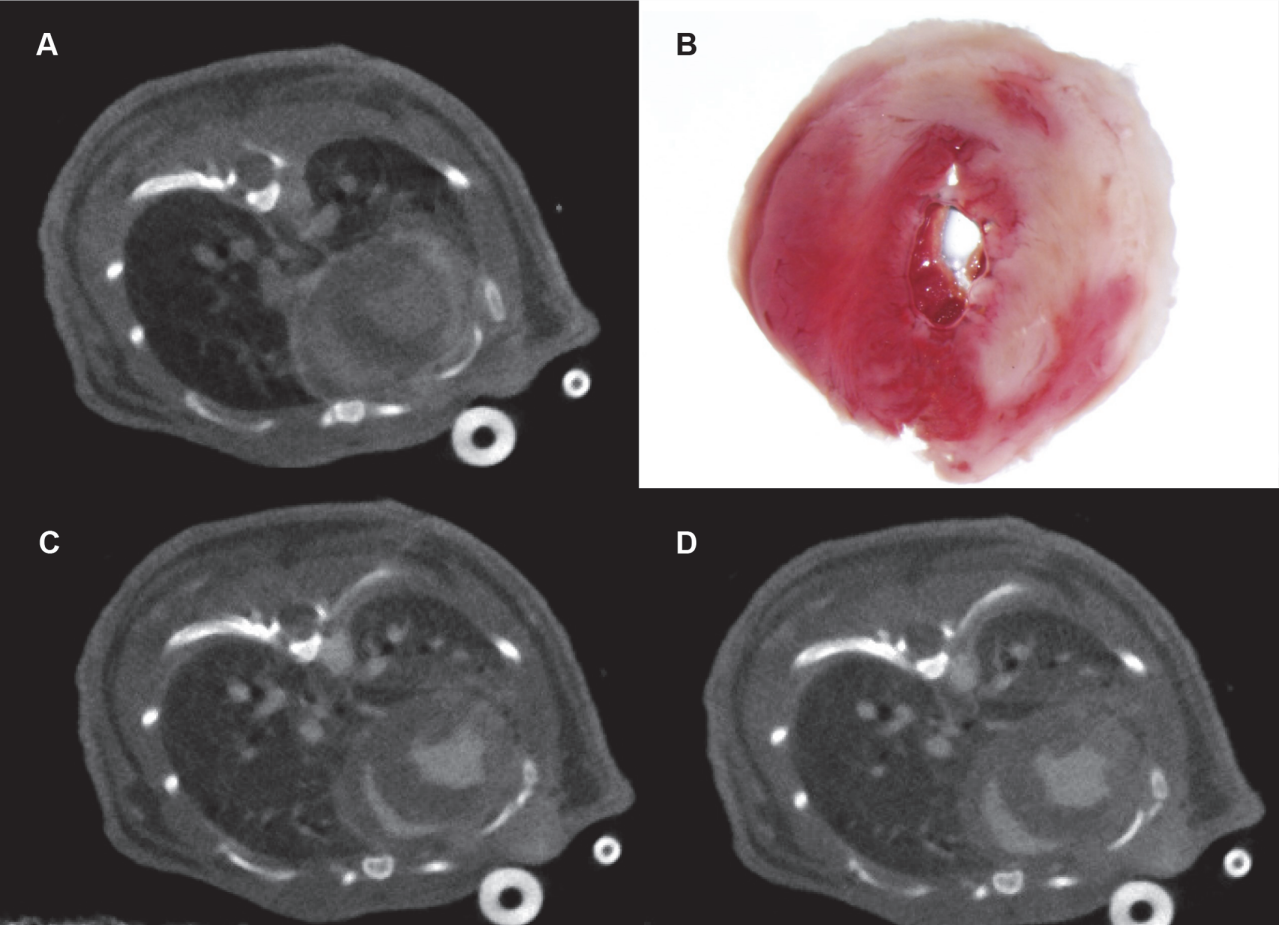
CT slices showing delayed contrast and LV volume, and matching histology. Areas of delayed iodine contrast enhancement in the infarcted myocardium are visible in micro-CT images (4A) due to contrast agent retention in fibrotic tissue. These portions of infarcted myocardial tissue appear on stained histological slices (4B). Indicators for infarcted myocardium are comparable in location, shape, and volume in both CT grayscale images and stained histological slices. At bottom, CT images of the same subject after administration of an iodinated lipid blood pool contrast agent. Images acquired during diastole (4C) and systole (4D) were used to calculate the ejection fraction.

A visual comparison was made between the delayed contrast micro-CT axial slices (Fig. 4A) and TTC-stained histological slices (Fig. 4B). Portions of infarcted myocardial tissue appear on TTC-stained histological slices as pale pink regions due to the lack of marker uptake. These TTC-stained axial heart slices clearly exhibit regions lacking protein uptake in the same regions of the myocardial wall, and are equivalent in size and shape, as those regions which display delayed contrast enhancement in micro-CT.

**Figure 5:**
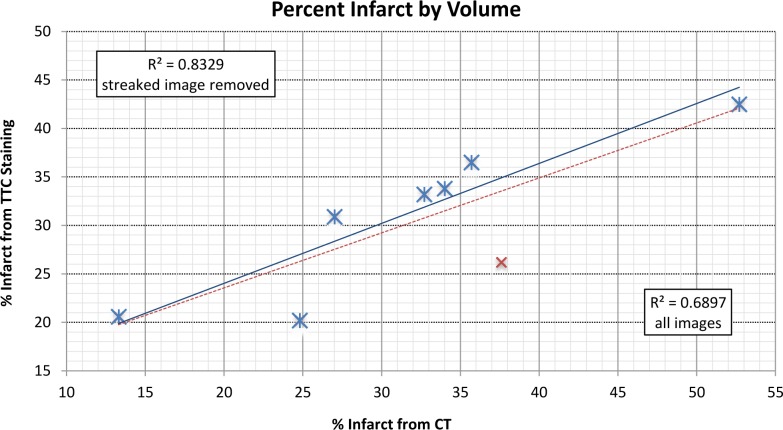
Comparison of percent infarcted tissue in left ventricle as derived by histology and CT. When results are plotted for all eight subjects, R^2^ = 0.6897. However, the CT-derived percentage was overestimated in subject #5 due to the presence of motion artifacts; when this subject is removed, R^2^ = 0.8329.

This relationship is quantified in Table 1; each subject’s percent volume of infarcted tissue is recorded, calculated separately by both CT axial slice and by histological TTC-staining analysis. Calculations from CT volumes estimate the average percent volume of infarcted tissue within the LV as 30.5 ± 7.8% while the analysis of histology gives the percent infarct as 32.2 ± 10.7% (similarity p < 0.71). The relationship between the values calculated by the two methodologies is plotted in Fig. 5. The CT image of subject ID #5 was obscured partially motion artifacts near the ribs arising from suboptimal physiological gating, leading to misattribution of these motion streaks as contrast hyper-enhancement by the segmentation algorithm. As a result, the percent infarct derived from this CT image could not be accurately determined. With the removal of subject ID #5, correlation of the two methodologies increased from R^2^ = 0.6897 to R^2^ = 0.8329.

**Table 1:** Infarcted volumes calculated as a percentage of the total left ventricle wall volume.

**Subject ID**	**CT Derived Infarct (%)**	**Histology Derived Infarct (%)**	**Difference (%)**
1	24.8	20.2	4.6
2	32.7	33.2	−0.5
3	13.3	20.6	−7.3
4	35.7	36.5	−0.8
5	37.6	26.2	11.4
6	34.0	33.8	0.2
7	27.0	30.9	−3.9
8	52.7	42.5	10.2
**AVERAGE**	**30.5**	**32.2**	
**ST. DEV.**	**7.8**	**10.7**	

Legend: Percent of infarcted tissue as derived from computed tomography images and from TTC-stained histological slices.

**Figure 6:**
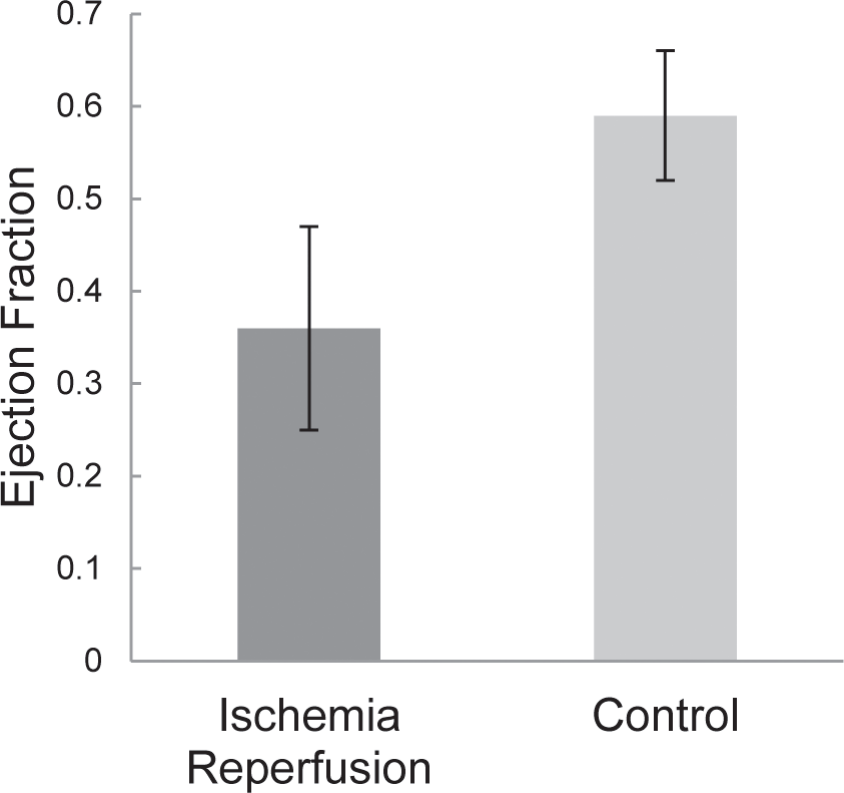
Calculated ejection fractions for ischemia reperfusion mice and controls. N = 7 for IR mice and N = 7 for controls. Error bars indicate one standard deviation.

In addition to the structural changes within the myocardium resulting from ischemia and reperfusion, micro-CT images provide information on heart function via ejection fractions, calculated from the images acquired during the diastolic and systolic cardiac phases (Fig. 6). The average calculated ejection fraction for the seven ischemia-reperfusion subjects was 0.36 ± 0.11 (n = 7) compared with 0.59 ± 0.7 (n = 7) for control subjects, quantifying a statistically significant difference in cardiac function resulting from the procedure (p < 0.01).

## Discussion

The results of CT image analysis and TTC-stained histological analysis confirm that the location, shape, and general size of the infarcted regions generally agree between modalities. While the average percent infarcted tissue from histological analysis is slightly greater than that derived by CT (32.2 ± 10.7% vs. 30.5 ± 7.8%), the difference may be partially explained by the non-isotropic spatial resolution of the images of the TTC-stained gross slices. Specifically, while reconstructed micro-CT images have a resolution of less than 80 microns in x, y, and z directions, photographing both the front and back of each 1mm TTC-stained slice results in an effective out-of-plane spatial resolution of only 500 microns. A possible result of this low out-of-plane resolution is an over-estimate of infarct volume reported by histology. Thinner slicing of harvested organs should improve accuracy but is practically challenging.

While the volume of infarcted tissue can be measured by tissue staining, measurement of the cardiac function of ischemia-reperfusion animals can only be determined with in-vivo imaging. Micro-CT imaging facilitated accurate LV blood pool measurements during diastole and systole, so that cardiac function could be quantified by ejection fraction. The values of ejection fraction calculated for healthy subjects and for subjects following ischemia reperfusion were quantifiably different and statistically significant, demonstrating the use of CNT micro-CT for such functional measurements.

The ability to identify infarcted tissue in the live animal is useful for longitudinal studies in mice. For example, infarct volume present immediately after surgery may increase or decrease through interventions. Without the ability to obtain high resolution images of infarcted tissue volume and distribution in vivo, the progress of an individual subject cannot be tracked, and many more mice would be required to evaluate an intervention statistically at intermediate time points. Despite the sensitive state of post-ischemic and reperfusion subjects, all study subjects survived 24 hours after surgery, and 7 of 8 subjects survived through the full imaging protocol. Given the noninvasive nature of the physiologically-gated micro-CT imaging protocol, longitudinal studies performed with the CNT micro-CT device are possible.

As with all physiologically-gated micro-CT imaging, the utility of data from the resulting images is dependent upon the quality of the gating protocol. While prospective gating is robust to many deviations from true periodic physiological motion, errors in gating due to respiratory and cardiac instability can lead to heart and rib motion blur, which can result in incorrect automatic segmentation and infarct volume measurements. Improvements in animal handling and increasingly sensitive physiological monitoring will enhance overall image quality and the accuracy of measurements.

Although prospective physiological gating and a free breathing protocol have many advantages, including minimized radiation dose and non-invasive handling, increased acquisition time results from the requirement of synchronizing both respiratory and cardiac motion for each x-ray projection. The ten to fifteen minute imaging time is on the same order of magnitude as the Iohexol contrast washout time, which poses a challenge in synchronizing imaging to the time-point of maximum iodine concentration in infarcted tissues. It would be optimal if the system scan time were reduced in order to allow acquisition of additional CT images prior to contrast washout. A reduction in total scan time could be achieved through a faster system gantry, one of the limitations in this prototype system. Alternatively, development of a contrast agent with a longer half-life with the correct properties to allow delayed contrast enhancement effects would have the same benefit as reducing scan time. Proposed improvements in the CNT micro-CT protocol, such as reducing the total number of projection images and applying a post-reconstruction bilateral filter to reduce Gaussian noise, could preserve image quality while simultaneously reducing scan time. Nonetheless, initial experimentation and the results of Nahrendorf et al [4] suggested that an efficient execution of our protocol would allow visualization of infarction within the optimal delayed enhancement time window, and our results confirm that the average scan time of 13 minutes was more than sufficient to capture the delayed enhancement effect with micro-CT.

The protocol of this study relied upon administration of two separate iodinated contrast agents; the long half-life lipid agent was administered only to guarantee sufficient contrast to perform LV volume measurements for ejection fraction. However, despite current hardware speed limitations, two CT image sets were able to be acquired during the half-life of Iohexol in the blood pool, and even images from the second acquisition exhibited sufficient contrast to measure LV blood pool volume. An amended procedure of sequential diastolic and systolic phase imaging immediately following Iohexol administration would shorten the study duration and eliminate the need for multiple contrast agents and resulting complications. Again, more rapid CT image acquisition would further facilitate the goal of single-contrast agent myocardial infarct imaging.

## Conclusions

The carbon nanotube micro-CT offers specific benefits over other imaging devices for in-vivo murine cardiac imaging. The short (15ms), high flux pulses produced by electronic triggering of the CNT cold cathode x-ray source allows a significant reduction in cardiac- and respiratory-motion blur. As this method of gated imaging does not require intubation and forced ventilation, it is minimally invasive and therefore appropriate for the most delicate of disease models (in particular a myocardial infarct model which must be imaged shortly after recovering from surgery). With CT images visualizing the delayed contrast effect, regions of myocardial infarct appear distinct from the surrounding uninjured tissue of the LV; these results compare in size, shape, and location to the regions of infarct indicated by TTC staining in histology. Furthermore, the pair of CT images obtained in diastole and systole allowed the measurement of ejection fractions, quantifying a significant decrease in heart function in subjects after ischemia and reperfusion. A delayed-enhancement contrast protocol, combined with the particular benefits of our novel CNT-cathode micro-CT and prospective respiratory and cardiac gating, provides a valuable new tool for the study of cardiac pathophysiology.

## References

[1] The International Journal of Public Health: media centre of cardiovascular diseases, the world health report 2012. Available: http://www.who.int/mediacentre/factsheets/fs317/en/index.html. Accessed 2013 December 10.

[2] Ordovas KG, Higgins CB (2011) Delayed Contrast Enhancement on MR Images of Myocardium: Past, Present, Future. Radiology 261(2): 358–374. 10.1148/radiol.11091882 22012903

[3] Sosnovik DE, Garanger E, Aikawa E, Nahrendorf M, Figuiredo JL, et al. (2009) Molecular MRI of Cardiomyocyte Apoptosis With Simultaneous Delayed-Enhancement MRI Distinguishes Apoptotic and Necrotic Myocytes In Vivo. Circulation: Cardiovascular Imaging 2:460–467. 10.1161/CIRCIMAGING.109.859678 19920044 PMC2780438

[4] Nahrendorf M, Badea C, Hedlund LW, Figueiredo JL, Sosnovik DE, et al. (2007) High-resolution imaging of murine myocardial infarction with delayed-enhancement cine micro-CT. Am J Physiol Heart Circ Physiol 292: H3172–H3178. 10.1152/ajpheart.01307.2006 17322414 PMC2680216

[5] Bartling SH, Stiller W, Grasruck M, Schmidt B, Peschke P, et al. (2007) Retrospective motion gating in small animal CT of mice and rats. Invest Radiol 42(10): 704–714. 10.1097/RLI.0b013e318070dcad 17984768

[6] Mahnken AH (2009) Assessment of Myocardial Edema by Computed Tomography in Myocardial Infarction. JACC Cardiovasc Imaging 2(10): 1167–1174. 10.1016/j.jcmg.2009.05.014 19833305

[7] Curley GF, Kevin LG, Laffey JG (2009) Mechanical ventilation: taking its toll on the lung. Anesthesiology 11: 701–703. 10.1097/01.anes.0000358753.29528.fb 19707117

[8] Zhang J, Cheng Y, Lee YZ, Gao B, Qiu Q, et al. (2005) A nanotube-based field emission x-ray source for microcomputed tomography. Rev Sci Instrum 76: 094301.

[9] Calderon-Colon X, Geng H, Gao B, An L, Cao G, Zhou O (2009) A carbon nanotube field emission cathode with high current density and long-term stability. Nanotechnology 20: 325707 10.1088/0957-4484/20/32/325707 19620758

[10] Qian X, Tucker A, Gidcumb E, Shan J, Yang G, et al. (2012) High resolution stationary digital breast tomosynthesis using distributed carbon nanotube x-ray source array. Med Phys 39: 2090 10.1118/1.3694667 22482630 PMC3321055

[11] Bordelon D, Zhang J, Graboski S, Cox A, Schreiber E, et al.(2008) A nanotube based electron microbeam cellular irradiator for radiobiology research. Rev Sci Instrum 79: 125102 10.1063/1.3043417 19123587 PMC2678784

[12] Cao G, Burk LM, Lee YZ, Calderon-Colon X, Sultana S, et al. (2010) Prospective-gated cardiac micro-CT imaging of free-breathing mice using carbon nanotube field emission x-ray. Med Phys 37: 5306 10.1118/1.3491806 21089765 PMC2951999

[13] Mukundan S (2006) A liposomal nanoscale contrast agent for preclinical CT in mice. AJR Am J Roentgenol 186(2): 300–307. 10.2214/AJR.05.0523 16423931

[14] Li HH, Du J, Fan YN, Zhang ML, Liu DP, et al. (2011) The ubiquitin ligase MuRF1 protects against cardiac ischemia/reperfusion injury by its proteasome-dependent degradation of phospho-c-Jun. Am J Pathol 178(3): 1043–1058. 10.1016/j.ajpath.2010.11.049 21356357 PMC3070562

[15] Yushkevich PA, Piven J, Hazlett HC, Smith RG, Ho S, et al. (2006) User-guided 3D active contour segmentation of anatomical structures: Significantly improved efficiency and reliability. Neuroimage 31: 1116–1128. 10.1016/j.neuroimage.2006.01.015 16545965

[16] Rich S, Chomka EV, Stagl R, Shanes JG, Kondos GT, et al. (1986) Determination of left ventricular ejection fraction using ultrafast computed tomography. Am Heart J 112: 392–396. 10.1016/0002-8703(86)90280-2 3739886

